# The study on the appearance of deformation defects in the yacht lamination process using an AI algorithm and expert knowledge

**DOI:** 10.1038/s41598-024-56410-w

**Published:** 2024-03-15

**Authors:** Paweł Szalewski, Tacjana Niksa-Rynkiewicz, Mariusz Deja

**Affiliations:** 1 Faculty of Ocean Engineering and Ship Technology, Institute of Naval Architecture, Gdańsk University of Technology, 11/12 Gabriela Narutowicza Street, 80-233 Gdańsk, Poland; 2 Faculty of Ocean Engineering and Ship Technology, Institute of Manufacturing and Materials Technology, Gdańsk University of Technology, 11/12 Gabriela Narutowicza Street, 80-233 Gdańsk, Poland

**Keywords:** Yacht, Lamination process, Algorithm A-priori, Deformation, Expert analysis, Production, Engineering, Mechanical engineering

## Abstract

This article describes the application of the A-priori algorithm for defining the rule-based relationships between individual defects caused during the lamination process, affecting the deformation defect of the yacht shell. The data from 542 yachts were collected and evaluated. For the proper development of the algorithm, a technological process of the yacht lamination supported by expert decisions was described. The laminating technology is a complex process of a sequential application of individual laminates according to a special strategy. The A-priori algorithm allowed for obtaining the set of association rules defining the relationships between the defects resulting from the lamination process and influencing the deformation defect of the yacht shell, which is one of the most common errors in yacht production. The obtained aggregated rules were compared with the expert knowledge of the employees of the production, quality control, mould regeneration, and technology departments of the yacht yard. The use of the proposed A-priori algorithm allowed for the generation of relationship rules consistent with the general opinion of experts. Associative rules additionally took into account detailed causes of a specific error, which were not always noticed by employees of specific departments. The assessment of the lamination process using an artificial intelligence algorithm turned out to be more objective, which made it possible to gradually reduce the total number of errors occurring in the yacht shell lamination process, and thus shorten the time needed to repair errors and the total time of producing the yacht.

## Introduction

The Polish yacht industry has been developing rapidly in last decades gaining high position in international markets^1^. Quality assurance at each manufacturing stage is a key factor for the competitiveness of yacht producers. Activities at the design and production stages can help to eliminate or reduce the number of defects, and thus shorten the time needed to manufacture the yacht. Since yachts are made of composite laminates, lamination is a key manufacturing process in the whole production. Laminates can be manufactured by the hand lay-up technique (HLU)^2^, the vacuum-assisted resin infusion (VARI) method^3^, and resin transfer molding (RTM)^4^. For glass fiber reinforced polymer laminates mechanical performance was similar after first two methods, but fewer local structure defects were observed for the samples obtained by VARI method^5^. The form of the lay-up and the outer 2–3 layers have the largest influence on the damage of composite marine structures under low-velocity impact^6^. The mechanical properties of laminates can be also predicted by the deformation theory of plasticity as shown on the example of ceramic composite laminates^7^. Apart from the lay-up scheme and mechanical properties, the geometry and support conditions have a significant impact on the vibro-acoustic behavior of laminated composite flat panels^8^.

Artificial intelligence techniques can be used to properly assess the quality of laminates. This allows to develop rules for the relationship between errors, which can be compared with the expert knowledge of experienced employees from various positions, i.e. quality controllers, laminate production workers, technologists, technicians involved in making moulds. Work on applying artificial intelligence to the production process based on intelligent and agile manufacturing^9^ has been ongoing for many years^10^.

Intelligent production of yachts can enable flexible and adaptive implementation of manufacturing operations, taking into account information technologies (IT), expert knowledge and, above all, artificial intelligence (AI) and intelligent computing^11^. Machine learning and the right algorithms can be used to better understand the production process and more accurately predict and avoid future problems, thus leading to improved and defect-free production. Deep reinforcement learning was used in^12^ to predict through inverse analysis shaft deformation by following stern hull deformation. The Deep Neural Network (DNN) predictive models were applied to predict the product quality in terms of average kerf in pulsed laser cutting of silicon steel sheet by utilizing vibration signals measured from the machine tool^13^. Data mining techniques using the A-priori algorithm were successfully used in^14^ to support the moulding machine maintenance. As a result, qualified association rules were extracted for predictive maintenance strategy applied to the manufacturing of wooden doors. The improved A-priori algorithm basing on Rough Set theory was developed in^15^ to search for quality association rules on the example of steel products. Identifying potential failure modes for product sub-systems and components was pointed out as a critical step in failure mode and effects analysis (FMEA). The A-priori algorithm can be used for exploring common sets of failure types to enhance product and system reliability^16^ and to extract frequent sequences of events that cause machines to fail. It can also be used to efficiently retrieve data from cloud data servers using metadata^17^ which in turn apply to cloud manufacturing^18^ based on huge quantity of information^19^.

The main emphasis in this work is on the problem of deformation of the yacht shell, which should be identified at the quality control stage and then removed. Although reverse engineering is successfully used at the quality control stage^20^, allowing for the proper assessment of difficult-to-see defects in the deformation of the yacht shell, the identification and location of some deformed planes require the work of an experienced employee. It turns out that it is very difficult to formalize expert knowledge regarding the causes of defects in the lamination process. Attempts to create a clear and transparent description of the defects themselves and the causes of their formation using only experts and measuring devices have not yet produced the expected results. Therefore, it is necessary to use additional tools based on continuous data exploration. Experts from yacht manufacturing companies confirm the need to develop a process of formalizing knowledge in this area, preferably in the form of rules that describe the relationships between errors occurring during the yacht lamination process. The construction of such a rule model may contribute to reducing the number of defects or even eliminating some defects occurring in the lamination process.

The A-priori algorithm and association methods presented in^21^ allowed for the formulation of a number of dependencies for the occurrence of defects in the lamination process in yacht production. Revealed relationships were not obvious to experienced technologists, but could occur with a high probability of coexistence. The obtained results allowed to shorten the total production time, but did not take into account the number of defects, and therefore the actual repair time, but only the occurrence of a specific defect.

This article presents a research method that allowed for the formalization of knowledge about the causes of deformation error during the yacht shell lamination process, using information about the number of observed errors. The A-priori algorithm allowed for obtaining aggregated rules which were compared with the expert knowledge of the employees of the production, quality control, mould regeneration, and technology departments of the yacht yard. The use of the proposed A-priori algorithm allowed for the generation of relationship rules consistent with the general opinion of experts. Associative rules additionally took into account detailed causes of a specific error, which were not always noticed by experts and employees of specific departments.

The reminder of this paper is organised in the following order. Section “Description of the research object” contains a brief description of the research object. In Section “Technological process of the yacht lamination supported by decision-making system”, the stages of the yacht lamination process supported by the decision-making system is reported. Sample defects caused in the lamination process and frequency of occurrence of single errors observed in the production of 542 yachts are also presented in this Section. The proposed research methodology is shown in Section “Research methodology”. Section “Results from the application of the A-priori algorithm” contains obtained results which were validated and compared with the experts knowledge in Section “Comparison of the results of the A-priori algorithm with expert knowledge”. The developed procedure and obtained results are discussed in Section “Discussion”. Finally, the authors’ approach and contributions are summarised in Section “Conclusions”.

## Description of the research object

For the research study, the CC6LA model of a laminated yacht was selected, with the general parameters presented in Table 1.

**Table 1:** General parameters of the CC6LA model of a laminated yacht used as a research object.

Yacht model	Length [m]	Width [m]	Immersion [m]	Total mass [kg]	Number of samples (Number of analysed yachts)
CC6LA	6.4	2.48	0.43	1036	542

**Figure 1:**
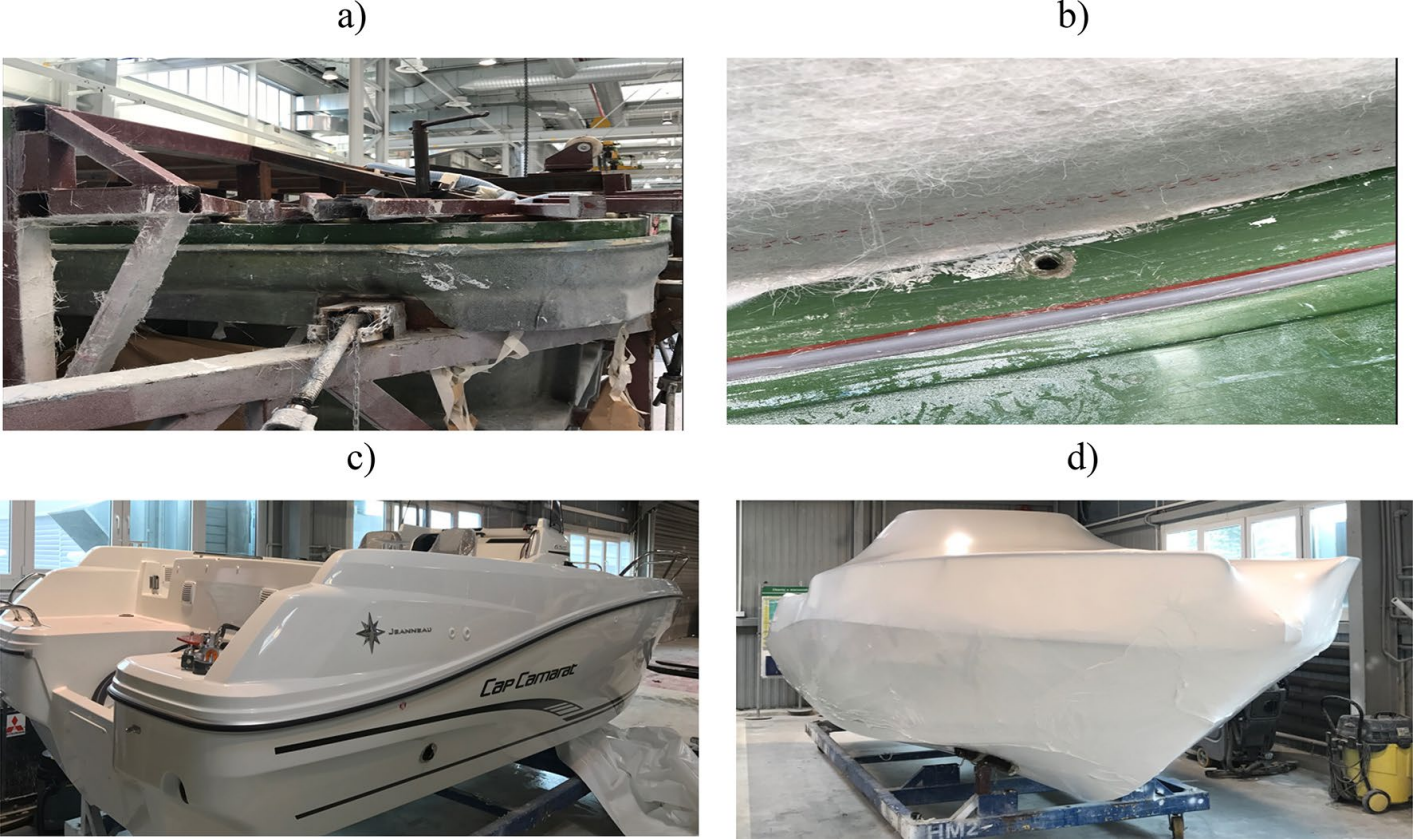
Selected stages of the yacht production: (**a**) preparation of the main mould, (**b**) lamination, (**c**) quality control, (**d**) preparation for shipping.

The data from the quality control after the preparation of the main mould (Fig. 1a) and the lamination process (Fig. 1b) were collected from 4 years. All yachts must meet high structural and aesthetic requirements during the quality control (Fig. 1c), so that the yachts can be shipped to the customer without the risk of complaints (Fig. 1d).

## Technological process of the yacht lamination supported by decision-making system

**Figure 2:**
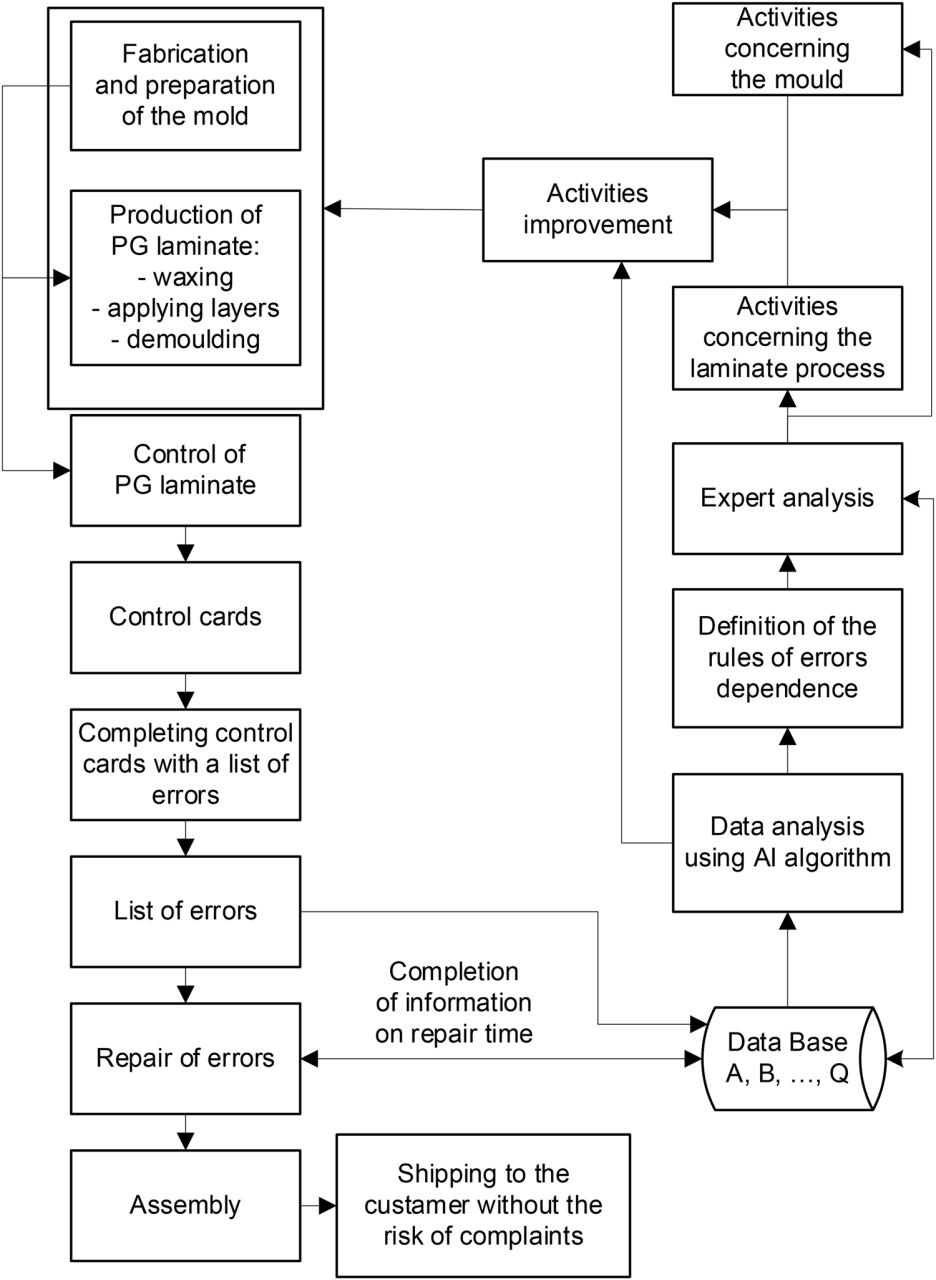
Stages of the yacht lamination process supported by AI methods and expert knowledge.

The stages of the yacht lamination process and supporting this process using artificial intelligence methods are presented in Fig. 2. The Technology Department is obliged to provide technical documentation necessary to make and prepare the mould (*Fabrication and preparation of the mould*). Making a mould begins with creating its metric containing the product type, product elements, mold number and manufacturer’s name. After creating the individual metric, the mould construction begins, including the preparation of a model on which subsequent layers of gelcoat and hardener are applied^22^. The form is strengthened and stiffened with additional structural elements. After checking the hardness of the gelcoat coating and the structural layers of the mould (the acceptance procedure) it is transferred to the production department of polyester-glass (PG) laminates.

**Figure 3:**
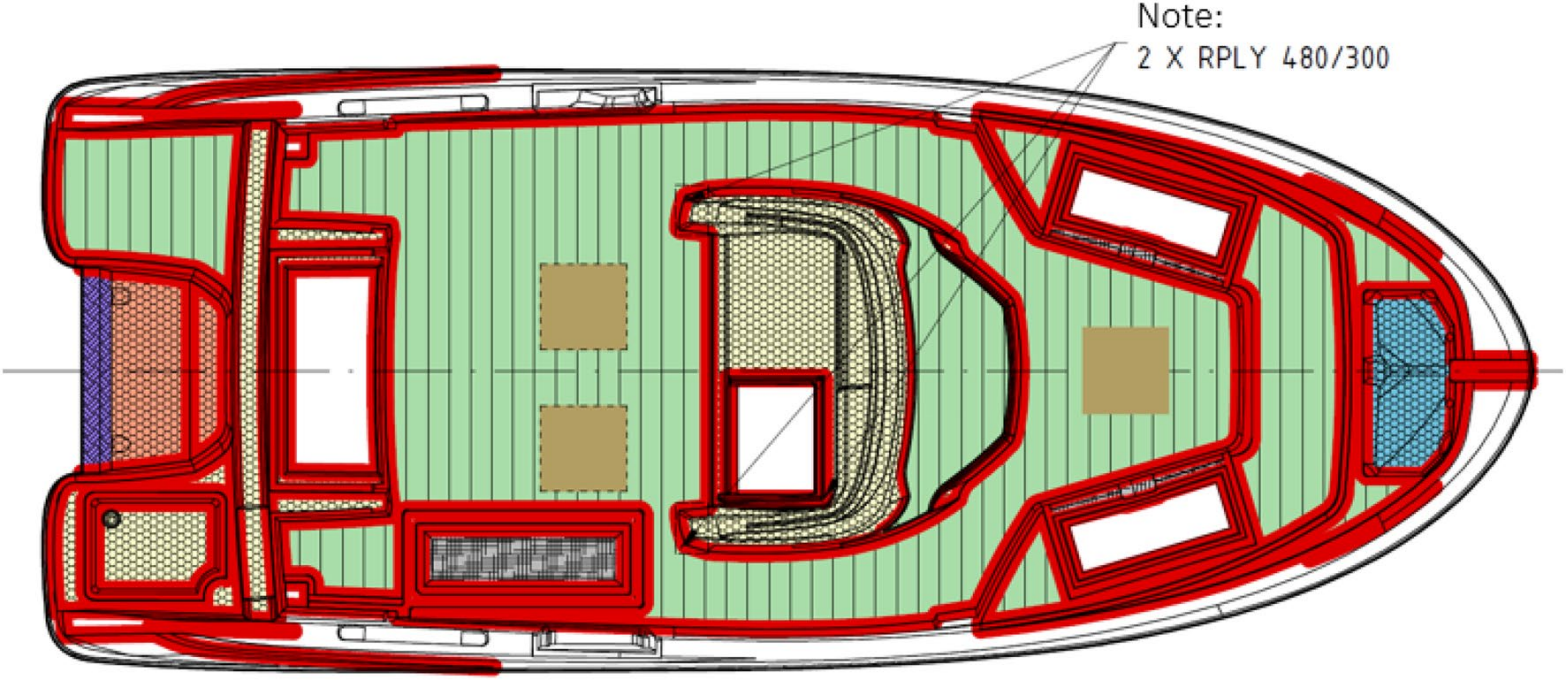
Scheme of applying different layers of laminates of the yacht deck on a previously prepared mould.

Making a PG laminate for a yacht hull begins with waxing and then applying individual layers of materials that make up the laminate. These activities must be carried out following the construction and technological documentation containing the number of layers, type, and weight of the material. The lamination process is not uniform for the entire product, as seen in the scheme of applying different layers of yacht deck laminate (Fig. 3).

Layers of different materials are applied at specific places according to a lamination code defined by color and number, following the sequence of layering presented in Table 2. The technological process for the exemplary laminates marked with seven colors and specific numbers requires 18 steps of layering and 2 technological breaks. It shows the complexity of the lamination process, which additionally must be carried out in controlled conditions taking into account temperature, humidity and air purity.

**Table 2:** The sequence of laying individual layers of laminate on a previously prepared mould using appropriate materials.

The sequence of layering	Lamination code defined by colour and number						
						
1	Gelcoat						
Technological break							
2	M 300	M 300	M 300	M 300	M 300	M 300	M 300
3	M 300	M 300	M 300	M 300	M 300	M 300	M 300
4	Edge reinforcement RPLY 480/300						
5			FIRET 2				
6			M 300	M 300			
7		TR 500	TR 500	TR 800		TR 500	
8		M 300	M 300	M 300		M 300	
9				FIRET 2			
10				M 300		M 300	
11				TR 800			
Technological break							
12	M 300				M 300		M 300
13	FIRET 2	AIREX 10			FIRET 2		
14	M 300	M 300			M 300		M 300
15	TR 500	TR 800			TR 800		TR 800
16					M 300		M 300
17					TR 800		TR 800
18					M 300		M 300

The PG laminate is inspected immediately after demoulding. The control card (Table 3) with a list of errors that can be identified after the lamination process is transferred to the repair department, where the number of errors (Quantity) is determined, expressed as the *Q*_*i*_ value for the *i*-th error, which affects the total repair time (*T*_*i*_). The preparatory time (*PREPA*) for repairing the defect, related to the collection of the necessary materials, such as sandpapers and gelcoats, as well as the unit times *t*_*ui*_ for repairing the error are downloaded from the *Database* and updated from time to time, resulting from the actual repair time. Thus total repair time *T*_*i*_ dedicated for the removal of the *i*-th defect is calculated as:

1 $${T}_{i}={PREPA}_{i}+\left({Q}_{i}\times {t}_{ui}\right)$$

where: *T*_*i*_—total repair time in minutes, *PREPA*_*i*_—preparatory time for repairing the defect in minutes, *Q*_*i*_—number of errors, *t*_*ui*_—unit time for repairing the defect in minutes.

**Table 3:** An exemplary standard control card with the list of defects and the repair time *T* for the sample yacht No. 193 of the CC6LA model.

No.	Defect ID	Short name of a defect	Name / description of a defect	$PREPA$	${Q}_{i}$	${t}_{ui}$	${T}_{i}$
1	A	Air bubbles	Air bubbles (of up 150 mm long or area similar to A5*)	10	21	6	136
2	B	Burnt surfaces	Burnt surfaces (area similar to A4*)	10	20	6	130
3	C	Surpluses to grind	Surpluses or loss of material—repair by grinding	10	4	6	34
4	D	Wrinkling	Wrinkling or “alligatoring”	10	3	6	28
5	E	Scratches	Small scratches (area similar to A4*) and scratches to be repaired	5	0	2	0
6	F	Cracks	Cracks (up to 250 mm long or area similar to A4*)	10	3	6	28
7	G	Inprint	Fibre Pattern (inprint)	5	0	6	0
8	H	Deformation	Deformation	**10**	**20**	**15**	**310**
9	I	Demould defects	Demould defects (up to 250 mm long or area similar to A4*)	5	25	4	105
10	J	Delamination	Delaminations and other construction laminate defects	10	0	15	0
11	K	Porosity	Porosity	10	0	11	0
12	L	Dimplings	Accelerator spots / Dimplings	10	0	6	0
13	M	Dry reinforcement	Dry glass reinforcements	10	0	15	0
14	N	Spots	Spots	10	0	6	0
15	O	Anti-sliding defects	Defects of Anti-sliding surface (300 mm^2^)	10	2	15	40
16	P	Matte finish	Matte finish	5	25	1	30
17	Q	Other defects	Other defects	0	0	30	0

*A4 and A5 are the size of the surface that corresponds to the size of the sheet of paper in the appropriate size.

**Figure 4:**
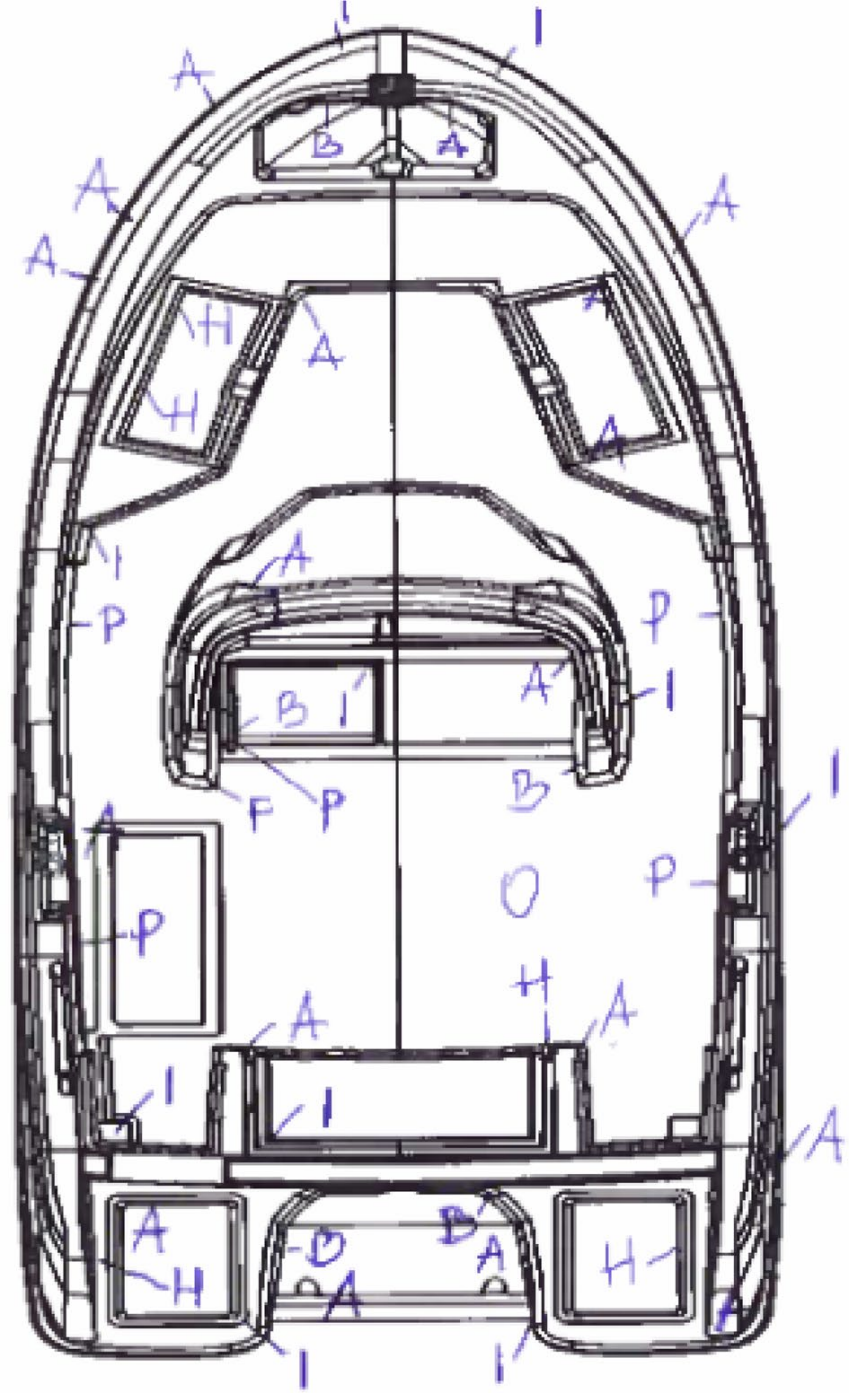
Defects marked by the technical staff during the quality control after the lamination process for the sample yacht No. 346: air bubbles (A), burnt surfaces (B), cracks (F), deformation (H), demould defects (I), anti-sliding defects (O), matte finish (P).

**Figure 5:**
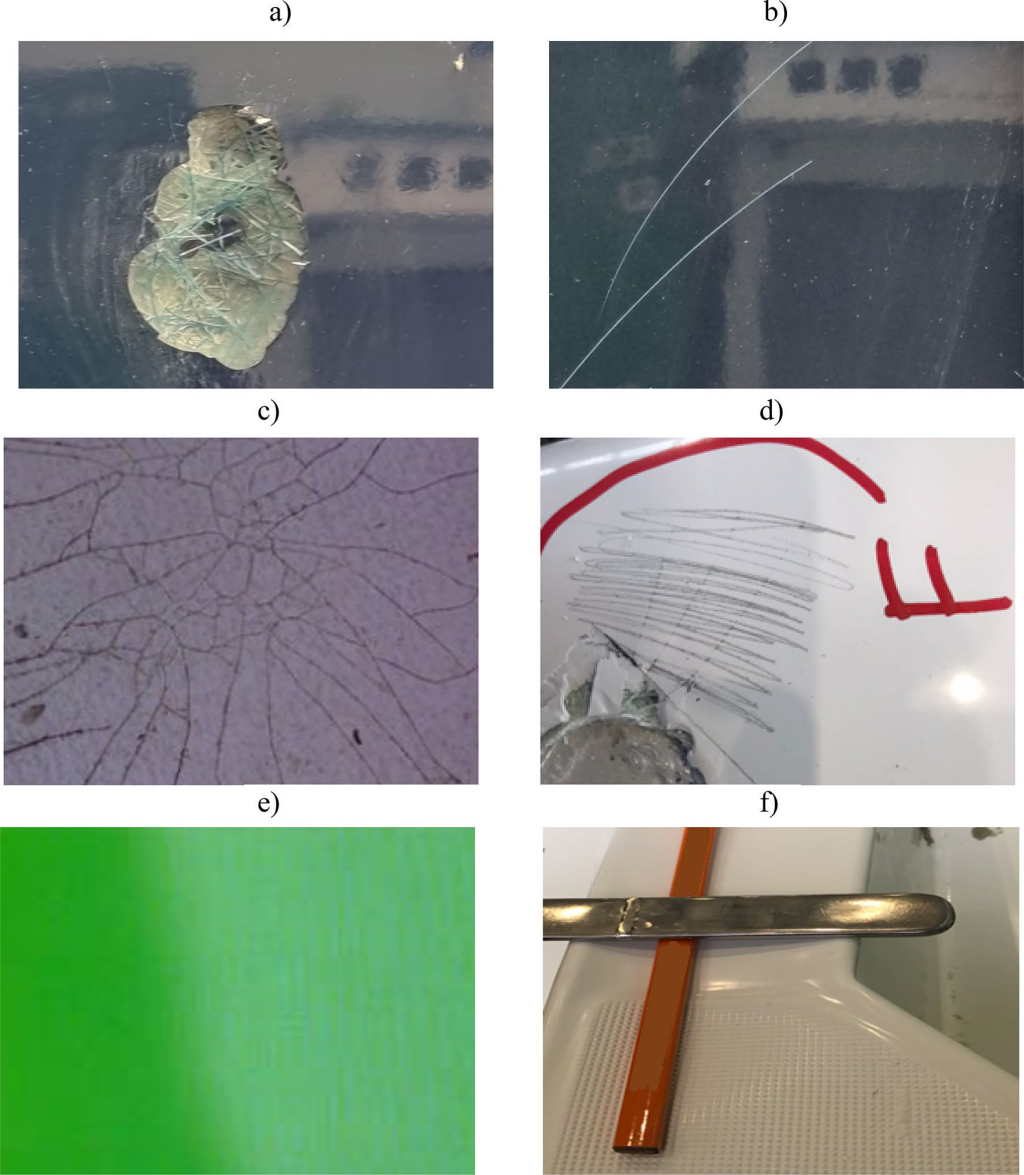
Sample defects caused in the lamination process: (**a**) surpluses to grind (defect C), (**b**) scratches (defect E), (**c**) cracks (defect F), (**d**) marking cracks that need to be repaired, (**e**) matte finish (defect P), (**f**) damage to anti-sliding (defect O) with concavity.

In addition to the number of errors, the places where they occur are also marked as shown in (Fig. 4). Images of sample errors occurring after the lamination process are shown in Fig. 5.

**Figure 6:**
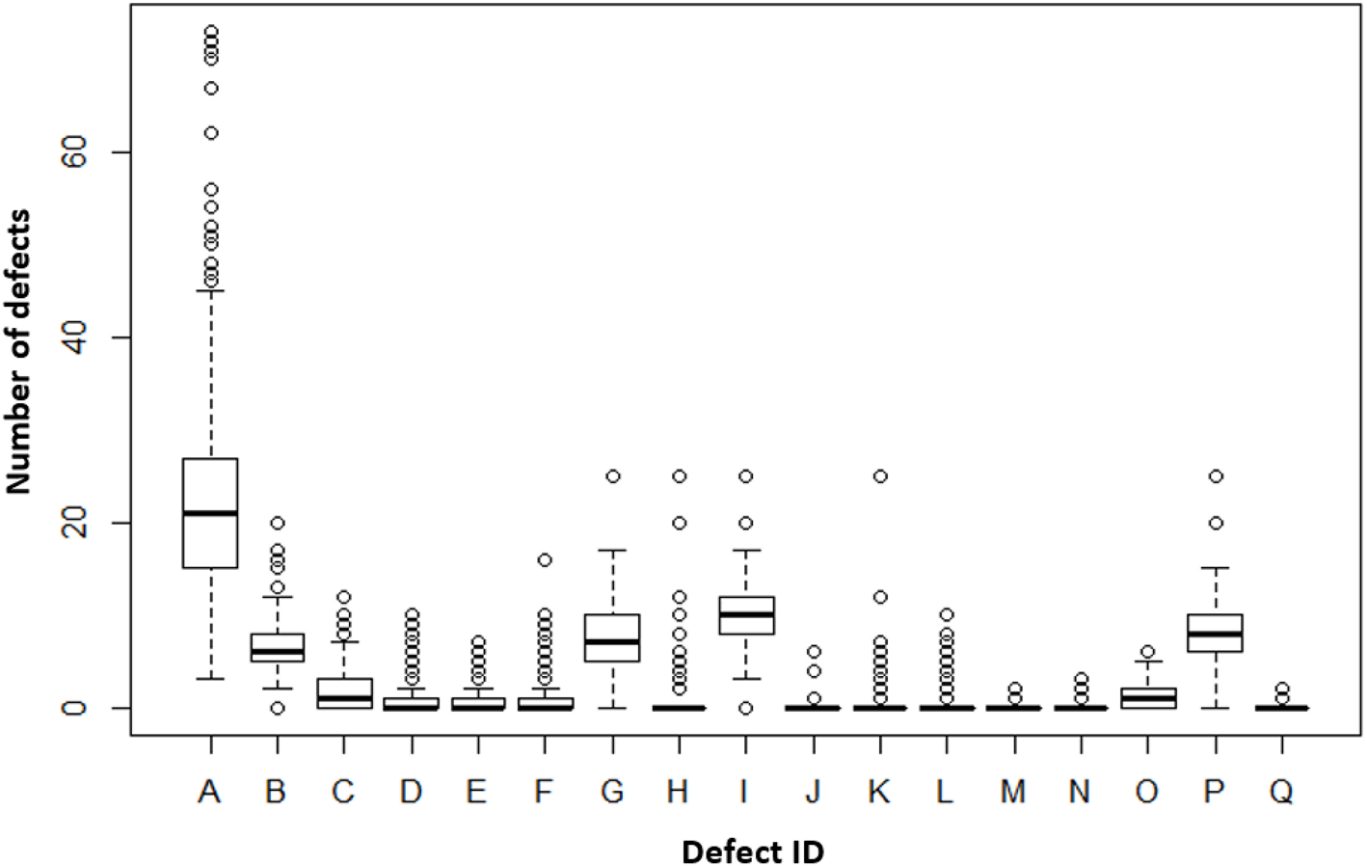
Boxplot of individual quantitative errors for all N-observations (N = 542).

The frequency of occurring individual errors observed in the production of N = 542 yachts is presented in Fig. 6. Although the H error is not the most common error (Fig. 6), the time needed to repair it is usually the longest (Table 3).

The statistical description of all errors is presented in Table 4. Analysis of the value of the first quartile (1st Qu) shows that 25% of the manufactured yachts had no more than 15 errors of A type. Error A appeared every time (from 3 to 73 air bubbles on one yacht). The remaining errors were not so notorious.

**Table 4:** List of errors and their characteristic values calculated for all defects and N-observations (N = 542).

Defect ID	Min	1st Qu	Median	Mean	3rd Qu	Max
A	3	15	21	22.47	27	73
B	0	5	6	6.054	8	20
C	0	0	1	1.747	3	12
D	0	0	0	0.9317	1	10
E	0	0	0	0.6919	1	7
F	0	0	0	0.8967	1	16
G	0	5	7	6.773	10	25
H	0	0	0	1.113	0	25
I	0	8	10	10.02	12	25
J	0	0	0	0.02768	0	6
K	0	0	0	0.2675	0	25
L	0	0	0	0.3266	0	10
M	0	0	0	0.02214	0	2
N	0	0	0	0.01661	0	3
O	0	0	1	1.338	2	6
P	0	6	8	7.965	10	25
Q	0	0	0	0.009225	0	2

## Research methodology

The proposed research method allows for the formalization of knowledge about the causes of the H deformation error during the lamination process, using information about the number of observed errors and, therefore, indirectly about the time needed to repair them. With the help of the A-priori algorithm, a database of rules describing the relationships between individual errors observed in the lamination process was built.

The advantage of the presented method is the ability to take into account the “air bubbles” error, which was not included in the methodology presented in^21^**,** but is one of the most common errors occurring during the lamination process, impossible to ignore in the opinion of technologists. In this research, authors improved the methodology by considering this very important defect A. According to the company standards, there is a minimum acceptable time which can be spent on the quality control and repairs, treated as a threshold for a defect qualification. If the number of defects is smaller, than the repair time is shorter and the defect may be qualified as a permissible and acceptable defect, not as a critical one.

In order to be able to effectively use information about the presence of error A for analysis, its quantitative assessment was introduced. It was based on the ratio of the number of type A defects to all observed *Q*_*c*_ errors. If error A constituted at least 15% of all errors, it was considered as a critical error and its occurrence was coded with the value 1. Below the 15% threshold, the error A was considered as an acceptable one and its occurrence was coded with the value 0 (Table 5). The value of 15% was determined in accordance with the industrial practice used in the analysed enterprise. As the production process is improved by introducing improvement activities, this value systematically decreases. Described procedure was applied to all 542 yachts and Table 5 contains the binary values 0 or 1 assigned to the defect A only for six exemplary yachts.

**Table 5:** Binary values of a defect A for exemplary six yachts; values 1 and 0 are for critical and permissible defects, respectively.

No.	Number of all defects (${Q}_{C}$)	0.15 *(${Q}_{C}$)	Number of type A defects	The binary value assigned to the error
1	67	10.05	28	1
2	87	13.05	10	0
3	133	19.95	39	1
4	124	18.6	26	1
5	53	7.95	19	1
6	56	8.4	21	1

Algorithm of discovering strong binary association rules *A-priori* was firs described in^23^. An association rule is based on the implication $\alpha \to \beta$ ; *If antecedent (*$\alpha$*) then consequent* $(\beta )$. The feature that distinguishes association rules from classic rules is the ability to assess their quality based on the indicators: support, confidence and lift.

The measure *support* $supp(\alpha \to \beta )$ is a measure for the $\alpha \to \beta$ rule and describes the relationship:

2 $$supp(\alpha \to \beta )=P(\alpha \cap \beta )$$

It is the ratio of the frequency of occurrence of records with *α* and *β* to all analysed transactions. The *confidence* $conf\left(\alpha \to \beta \right)$ measure tells about the conditional probability of the rule occurring:

3 $$conf(\alpha \to \beta )=P(\alpha \cap \beta )/P(\alpha )$$

The *lift* $lift(\alpha \to \beta )$ measure is a measure of rule importance, using both *support* and *confidence* measures:

4 $$lift(\alpha \to \beta )=\frac{P(\alpha \cap \beta )}{P\left(\alpha \right) * P(\beta )}$$

The *lift* measure tells how much the probability of the event $P(\alpha \cap \beta )$ is greater than the probability of events $P(\alpha )$ and $P(\beta )$ occurring independently of each other. As the *lift* index increases, the quality of the rule increases. The A-priori algorithm was used to discover the most frequently occurring event sets.

### Ethical approval and Informed consent

All experimental protocols and written surveys were approved by the management board of Ostróda Yacht Sp. z o.o. Informed consent was obtained from all subjects and their legal guardians.

### Guideline statement

All methods were carried out in accordance with relevant guidelines and regulations.

## Results from the application of the A-priori algorithm

The A-priori algorithm was run with the assumption that consequent refers to the H defect, having at its disposal the set of observations described in the previous section. Taking into account that the observed errors may appear rarely, but always in the same correlation, the following coefficient values were assumed: $supp=0.1$, $conf=0.1$. For the assumptions adopted in this way, 73 rules were obtained, but only those rules for which the value of the *lift* parameter was significant, i.e. $lift>2$, were selected for analysis. In this way, the most significant relationships were nominated, the presence of which may correlate with the appearance of deformation error (Table 6).

**Table 6:** List of rules $\alpha \to \beta$ where $\beta =H$ and $lift>2$ nominated for further analysis.

Rule no.	$\alpha$	Lift	Count
1	{B, C, F, I, O}	2.751759	57
2	{B, C, F, I, O, P}	2.751759	57
3	{C, F, I, O, P}	2.745462	60
4	{B, C, F, O}	2.726743	57
5	{B, C, F, O, P}	2.726743	57
6	{C, F, O, P}	2.721794	60
7	{C, F, I, O}	2.721794	60
8	{C, F, O}	2.698531	60
9	{F, I, O, P}	2.545085	74
10	{B, F, I, O, P}	2.540341	70
11	{F, O, P}	2.528559	74
12	{B, F, O, P}	2.522942	70
13	{B, F, I, O}	2.522942	70
14	{F, I, O}	2.512246	74
15	{B, F, O}	2.505779	70
16	{F, O}	2.496141	74
17	{B, C, F, I}	2.192557	60
18	{C, F, I, P}	2.189614	62
19	{B, C, F, I, P}	2.186380	59
20	{B, C, F}	2.177436	60

Based on the computations performed, it was noticed that for two-element frequent sets there is a strong relationship only between the deformation error (H) and cracks F up to 250 mm (Fig. 5c), and damage to anti-sliding O (Fig. 5f). This relationship is determined by a rule created on the basis of inference from two-element frequent sets. The rule is in the form:

5 $$IF \; F \; AND \; O \; THEN \; H \; (lift: 2.5)$$

A more complex relationship was constructed based on the aggregation of rules from three-element frequent sets:

6 $$If \;F \;AND\; O \; AND \; (\{C\} \; OR \; \{P\} \; OR \{I\} \; OR \{B\}) \; THEN \; H \; (lift: 2.6)$$

And based on the aggregation of rules from four-element frequent sets, the following formula was constructed:

7 $$IF \; F \; AND \; O \; AND \; C \; AND \; ( \{P\} \; OR \; \{I\} \; OR \; \{B\}) \; THEN \; H \; (lift: 2.7)$$

The method used also allowed to identify and formalize the strong dependence of deformation (H) on the crack error up to 250 mm (F) and the burnt surfaces error (B). The resulting rule can be written as:

8 $$IF \; B\; AND \; F \; AND \; (\{O\} \; OR \; \{C\}) \; THEN \; H \; (lift: 2.3)$$

**Figure 7:**
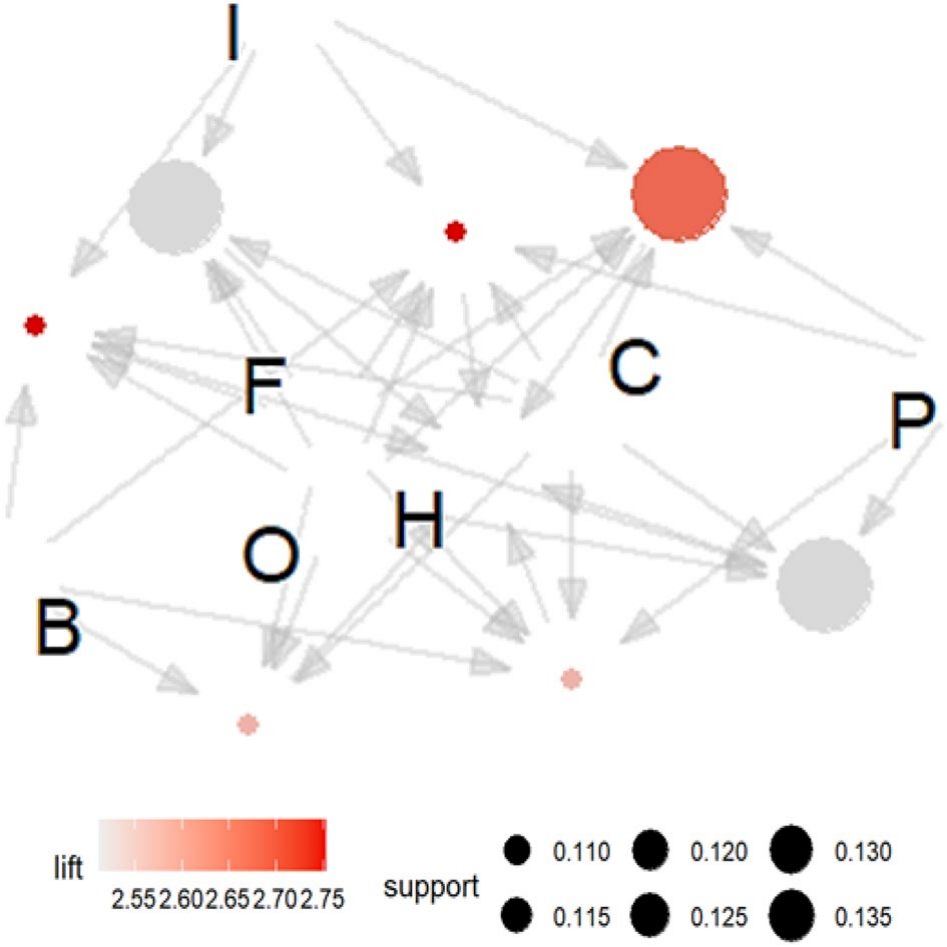
Visualization of the relationship between errors that have a direct impact on deformation (H), along with information about the values of support and lift indicators.

**Figure 8:**
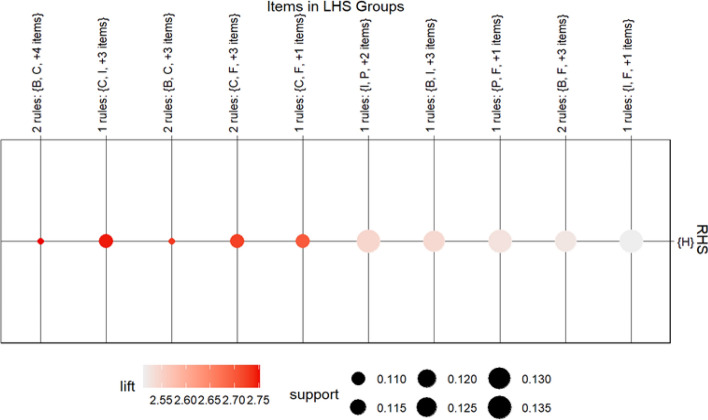
Deformation error dependency grid (H) as a successor of rules and their antecedent (Items in LHS Groups) including information about the values of *support* and *lift* indicators.

According to the rule described by Eq. (7), the dependence of the deformation error H on F and O strengthens the presence of errors P, I or B. Similarly, according to the rule described by Eq. (8), the dependence of the deformation error H on B and F strengthens the presence of error O or C. The rules resulting from analyses of five-element frequent sets and their graphical interpretations (Figs. 7 and 8) show that the strongest influence on the deformation error is exerted by errors B, C, F, I, O, P. The rules that describe this relationship have higher values of the *lift* and *support* indicators.

The analysis of the collected observations allowed to additionally detect a negative correlation between errors A and H ($lift<1$). H error usually occurs when there is a smaller amount of A error or $\{A,I\}$ errors (Table 7).

**Table 7:** List of rules $\alpha \to \beta$, where $\beta = H$ and $lift < 1$.

Rule no.	$\alpha$	Lift	Count
1	{A}	0.8875892	56
2	{A, I}	0.8770227	55

Three relationships were noticed for the opposite case when trying to find rule consequents with the antecedent H (Table 8). With the same initial assumptions, the algorithm generated 8 more rules. None of them had a $lift > 2$.

**Table 8:** List of rules $\alpha \to \beta$, for $\alpha =H$.

Rule no.	$\alpha$	$\beta$	*Lift*	Count
1	H	F	1.8672095	77
2	H	D	1.6444175	60
3	H	O	1.5341504	100

## Comparison of the results of the A-priori algorithm with expert knowledge

The rules generated using the A-priori algorithm, presented in Table 9 and described in Eqs. (5)–(8), were compared with the opinions of experts from four departments of the company: production, quality control, mould regeneration, and technology. The expert questionnaires were completed without knowledge of the results obtained from the applied computational procedure. The impact of each defect on the H error was assessed by experts on a scale from 0 to 100%, where 100% meant the greatest impact.

**Table 9:** Two-, three-, and four-element rules selected for comparison with expert knowledge.

Rule symbol	R1	R2	R3	R4	R5	R6	R7	R8	R9
Rule	{F,O}	{F,O,C}	{F,O,P}	{F,O,I}	{F,O,B}	{B, F, C}	{F,O,C,B}	{F, O, C, I}	{F, O, C, P}

**Figure 9:**
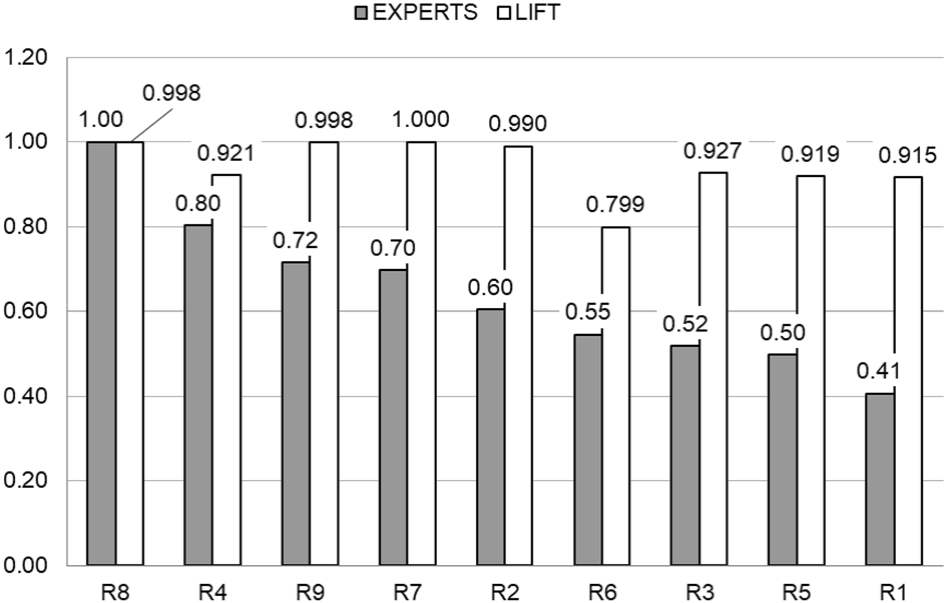
The influence of two-, three- and four-element rules R1–R9 on the deformation error H according to expert opinions and the *lift* indicator.

After completing 50 questionnaires, the results were normalized to be between 0 and 1—Fig. 9. According to experts, the greatest impact on the deformation error H has the defects occurring in the four-element rule R8 and the three-element rule R4, i.e. in the group of defects F, O, I (R4) with defect C (R8). Next, the defects included in the four-element rules R9 and R7 were classified, containing defects F, O, C from group R2, with defect B (R7) or P (R9). The three-element rules R6, R3 and R5 and the two-element rule R1 were classified the lowest. These rules also obtained lower values of the normalized *lift* coefficient—Fig. 9. Similarly, the rules R8, R9, R7 and R2, highly classified by experts, have higher values of the *lift* coefficient, close to or equal to 1. A lower value of the *lift* coefficient was obtained by the three-element rule R4, highly rated by experts, which is, however, included in the R8 rule, highly rated both by experts and the A-priori algorithm.

**Figure 10:**
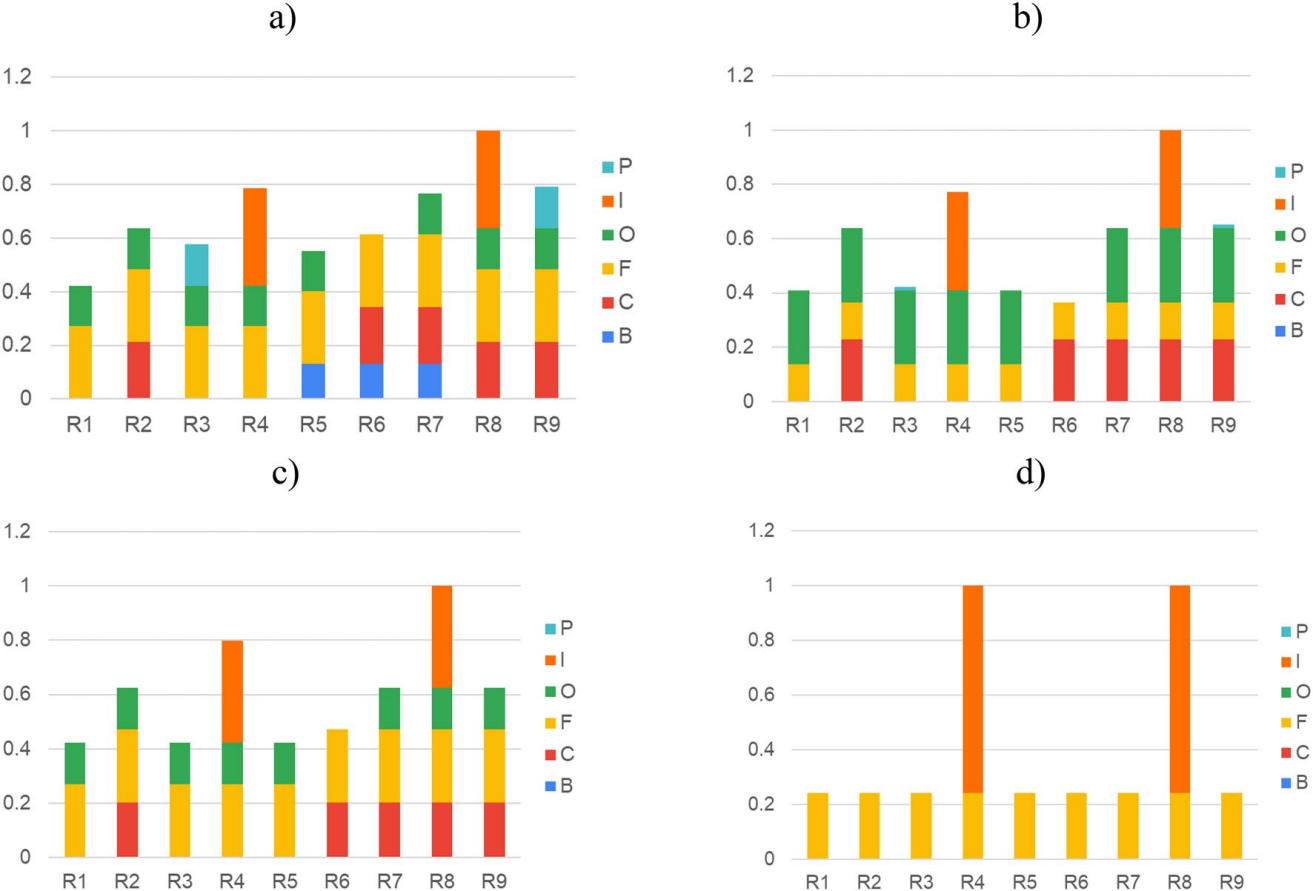
The impact of error components contained in rules R1–R9 on the deformation error H according to the opinions of experts from various yacht company departments: (**a**) production, (**b**) quality control, (**c**) mould regeneration, (**d**) technology.

The greatest impact of the R4 and R8 rules on the deformation error H indicated in all expert surveys (Fig. 9) was also noticed and confirmed in the surveys completed by employees of specific departments – Fig. 10. However, the impact of the error components of specific rules is perceived differently by employees of different departments. Experts from the production department, i.e. department directly involved in the process of manufacturing the laminate shell, indicated in the surveys that all 6 defects P, I, O, F, C, B from the R1–R9 relationships deduced using the AI algorithm have an impact on the deformation error H – Fig. 10a.

Experts from the quality department indicated the impact of five defects P, I, O, F, C, with a minimal impact of defect P and no impact of defect B—Fig. 10b. Experts from the mould regeneration department indicated the impact of four defects I, O, F, C, with no influence of defects P and B—Fig. 10c. However, experts from the technology department, having the least contact with production, indicated the influence of only two defects I and F on the deformation error H—Fig. 10d.

## Discussion

The proposed research method allowed for the formalization of knowledge about the causes of the H deformation error during the lamination process, using information about the number of observed errors. The A-priori algorithm allowed for obtaining aggregated rules defining the relationships between the defects resulting from the lamination process and influencing the deformation defect of the yacht shell, which is the most common defect in the yacht production.

The presented results confirm the validity of the rule inference algorithm used, and also indicate the advisability of its application in a company where production is based on significant human participation at every stage of production, i.e. at the design, manufacturing, quality control and repair. This is influenced by, among others, different levels of expert knowledge and professional experience of people employed in individual departments. The division of expert surveys into the following departments: production, quality control, mould regeneration and technology, showed different perceptions by their employees of the impact of individual defects, which are components of aggregated rules generated using the A-priori algorithm, on the deformation error. The use of the proposed A-priori algorithm allowed for the generation of dependency rules consistent with the general opinion of experts, but taking into account detailed causes of a specific error, not always noticed or even ignored by employees of specific departments. This makes the assessment using an artificial intelligence algorithm more objective, enabling, through the introduction of technological improvements, to gradually reduce the total number of errors occurring in the yacht shell lamination process, and thus shorten the time needed to repair errors and the total time of producing the yacht.

## Conclusions

The main contributions and benefits of this work can be summarised as:

- The proposed methodology allowed for the formalization of knowledge about the causes of defects in the yacht lamination process;
- The A-priori algorithm allowed for obtaining aggregated rules defining the relationships between the defects resulting from the lamination process and influencing the deformation defect of the yacht shell;
- The generated dependencies turned out to be consistent with the general opinion of experts, but they also took into account more detailed causes of a specific error, not always noticed or even ignored by employees of specific departments.
- The developed methodology can be applied in companies with various production profiles, especially those based on significant human participation at every stage of the production, i.e. at the design, manufacturing, quality control and regeneration.
- Assessment of the laminating process using an artificial intelligence algorithm turned out to be more objective due to employees’ different perceptions of individual errors and relationships between specific defects. This is influenced by, among others, different levels of knowledge and professional experience of people employed in individual departments.

## Data Availability

The data that support the findings of this study are available from the corresponding author, [MD], upon reasonable request.
